# 

**Published:** 1875-07

**Authors:** 


### CONTENTS:

### ORIGINAL COMMUNICATIONS.

ARTICLE I.—Prevention and Treatment of Decay on Proximal Surfaces of the Teeth. By G. H. Cushing, D. D. S. .......... 97
ARTICLE II.—South Carolina State Dental Association .......... 116
American Dental Convention .......... 117
American Dental Association .......... 118
Massachusetts Dental Society .......... 119

### SELECTED ARTICLES.

ARTICLE III.—Salicylic Acid .......... 120

ARTICLE IV.—Partial Exsection of both Upper Jaws for Enchondroma .......... 126

ARTICLE V.—Deleterious Results from Wearing Artificial Teeth Set Upon the Continuous Rubber Base .......... 129

ARTICLE VI.—Hemorrhagic Diathesis .......... 131

ARTICLE VII.—Amalgams .......... 134

### EDITORIAL, ETC.

Letter from Dr. Nash .......... 135
A Medical Imbroglio .......... 136
Celluloid Manufacturing Company .......... 138
A Tooth in the Tongue .......... 140

### OBITUARY.

Dr. O. N. Allan .......... 141

### MONTHLY SUMMARY.

The Diagnosis of the Lead-line .......... 142

Cold Powder .......... 142

Lead Poisoning in Lead Workers .......... 143

Thymol an Antiseptic and Antifermentative Substance .......... 143

Case of Abstention from Food .......... 144

